# Prototyping the implementation of a suicide prevention protocol in primary care settings using PDSA cycles: a mixed method study

**DOI:** 10.3389/fpsyt.2024.1286078

**Published:** 2024-01-25

**Authors:** Nadia Minian, Allison Gayapersad, Adina Coroiu, Rosa Dragonetti, Laurie Zawertailo, Juveria Zaheer, Braden O’Neill, Shannon Lange, Nicole Thomson, Allison Crawford, Sidney H. Kennedy, Peter Selby

**Affiliations:** ^1^INTREPID Lab, Centre for Addiction and Mental Health, Toronto, ON, Canada; ^2^Department of Family and Community Medicine, University of Toronto, Toronto, ON, Canada; ^3^Institute of Medical Sciences, Faculty of Medicine, University of Toronto, Toronto, ON, Canada; ^4^Department of Pharmacology and Toxicology, University of Toronto, Toronto, ON, Canada; ^5^Campbell Family Mental Health Research Institute, Centre for Addiction and Mental Health, Toronto, ON, Canada; ^6^Institute for Mental Health Policy Research, Centre for Addiction and Mental Health, Toronto, ON, Canada; ^7^Department of Psychiatry, University of Toronto, Toronto, ON, Canada; ^8^Institute of Health Policy, Management and Evaluation, University of Toronto, Toronto, ON, Canada; ^9^Arthur Sommer Rotenberg Program in Suicide Studies, Unity Health Toronto, Toronto, ON, Canada; ^10^Dalla Lana School of Public Health, University of Toronto, Toronto, ON, Canada

**Keywords:** suicide prevention, primary care, implementation, CFIR, ERIC, PDSA

## Abstract

**Introduction:**

In Canada, approximately 4,500 individuals die by suicide annually. Approximately 45% of suicide decedents had contact with their primary care provider within the month prior to their death. Current versus never smokers have an 81% increased risk of death by suicide. Those who smoke have additional risks for suicide such as depression, chronic pain, alcohol, and other substance use. They are more likely to experience adverse social determinants of health. Taken together, this suggests that smoking cessation programs in primary care could be facilitators of suicide prevention, but this has not been studied.

**Study objectives:**

The objectives of the study are to understand barriers/facilitators to implementing a suicide prevention protocol within a smoking cessation program (STOP program), which is deployed by an academic mental health and addiction treatment hospital in primary care clinics and to develop and test implementation strategies to facilitate the uptake of suicide screening and assessment in primary care clinics across Ontario.

**Methods:**

The study employed a three-phase sequential mixed-method design. Phase 1: Conducted interviews guided by the Consolidated Framework for Implementation Research exploring barriers to implementing a suicide prevention protocol. Phase 2: Performed consensus discussions to map barriers to implementation strategies using the Expert Recommendations for Implementing Change tool *and* rank barriers by relevance. Phase 3: Evaluated the feasibility and acceptability of implementation strategies using Plan Do Study Act cycles.

**Results:**

Eleven healthcare providers and four research assistants identified lack of training and the need of better educational materials as implementation barriers. Participants endorsed and tested the top three ranked implementation strategies, namely, a webinar, adding a preamble before depression survey questions, and an infographic. After participating in the webinar and reviewing the educational materials, all participants endorsed the three strategies as *acceptable/very acceptable* and *feasible/very feasible*.

**Conclusion:**

Although there are barriers to implementing a suicide prevention protocol within primary care, it is possible to overcome them with strategies deemed both acceptable and feasible. These results offer promising practice solutions to implement a suicide prevention protocol in smoking cessation programs delivered in primary care settings. Future efforts should track implementation of these strategies and measure outcomes, including provider confidence, self-efficacy, and knowledge, and patient outcomes.

## 1 Introduction

Suicide is a serious public health issue, causing significant burden to individuals, families, and the healthcare systems (1–4). Despite the fact that the suicide mortality rates have largely been decreasing over time (24% between 1981 and 2017) (5, 6), approximately 4,500 people die by suicide in Canada every year (7, 8). The suicide rate in Canada in 2019 was 12.1 per 100,000 population; however, there are notable differences in the rate across provinces and territories, as well as across the sexes (9). Historically, compared with women, suicide rates among men are three to four times higher (6, 10). Given its population size, Ontario accounts for approximately 35% of Canadian suicides (8). While an in-depth exploration is beyond the scope of this study, studies have demonstrated that although there is no clear evidence for a causal effect, there is a strong association between current cigarette smoking and suicide-related behaviors (11–14). Current versus never smokers have an 81% increased risk of death by suicide (11). Those who smoke often have additional risks for suicide, such as depression, alcohol and other substance use, and chronic pain (11, 12, 15). Moreover, they are more likely to experience adverse social determinants of health (16).

For the purposes of this study, while the authors acknowledge that there is much debate and agreed upon nomenclature and classification for suicidal ideation and behavior (17), the terms used will follow the definitions^1^ provided by Silverman et al. (18–20). Suicide ideation is a predictor of suicide attempt, especially when accompanied by a clearly delineated plan and the means to carry out the plan (21). On average, 2.5% of Canadians report suicidal ideation during the past year (7). Notably, 95% of people who attempt or die by suicide also report visiting a family physician in the previous year (7), and approximately 45% of people who die by suicide have had contact with their primary care provider (compared with 19% who visited a mental health professional) within 1 month of their suicide (2). This suggests a window of opportunity for suicide prevention in primary care. There is strong evidence showing that suicide prevention interventions are effective in reducing suicide attempts and increasing treatment initiation (22, 23). By conceptualizing suicide within the ideation-to-action framework (21, 24), it is reasonable to posit that the prevention of suicide can be accomplished, at least in part, by addressing suicidal ideation.

Systematic approaches to suicide prevention in primary care could mitigate suicide risk (25–27). Primary care settings are an important and often underutilized resource to help prevent suicides, as they provide longitudinal, relationship-based care for acute and chronic illnesses and have numerous contact points with patients to deliver suicide prevention interventions (28–31). Effective and scalable interventions for suicide prevention exist (26, 32, 33). Recent research has shown that brief, one-time, suicide prevention interventions in emergency departments are associated with a reduction in subsequent suicide attempts (22) and can easily be delivered in primary care (3). Thus, it is not surprising that suicide prevention is a core competency for Canadian family physicians (34). However, implementation of suicide prevention protocols in primary care is often lacking (35). While universal (i.e., of the general population) screening for suicide ideation is not supported by current evidence (36–38), screening of the high-risk groups, including people with mental health and substance use disorders and people who smoke, is generally regarded as standard of care (12, 39, 40).

In 2018, as part of a cluster-randomized trial comparing two approaches for delivering evidence-based mood management interventions within an existing smoking cessation program (41), several members of our team developed and implemented a suicide risk assessment protocol to assist non-clinical research staff with identifying and triaging individuals at risk of suicide. The protocol used the Patient Health Questionnaire (PHQ-9) (42) to assess patients’ mood. To develop the protocol, we took the social determinants of health and their role in suicide ideation into consideration. First, in 2018, nine research assistants (RAs) in the Smoking Treatment for Ontario Patients (STOP) program at the Center for Addiction and Mental Health (CAMH), the largest mental health teaching hospital in Canada, were trained and tasked with implementing the protocol (an additional 7 research assistants received training to implement the protocol between September 2018 and March 2020). Second, in 2020, we shared the protocol with healthcare providers (HCPs) in primary care, who are part of the STOP program. The STOP program is an established smoking cessation program implemented in healthcare organizations across Ontario, Canada, which offers up to 26 weeks of smoking cessation treatment (nicotine replacement therapy and behavioral counseling) at no cost to the patients (43). In case of the other settings (35, 44), the implementation of this protocol into practice has been inadequate.

Research has indicated that factors related to clinicians, such as their knowledge and experience in dealing with patients at risk for suicide, as well as factors associated with the treatment setting, such as the geographical location, can influence the utilization of suicide prevention protocols (45). The goals of this study were to (1) understand the barriers to and facilitators of implementing evidence-based suicide prevention protocols from the perspective of both RAs and HCPs and (2) develop and test implementation strategies to facilitate evidence-based suicide screening, assessment, and intervention in team-based primary care clinics in Ontario.

## 2 Methods

### 2.1 Design

This study employed a three-phase mixed-method sequential exploratory design (46). Phase 1 included qualitative semi-structured interviews to identify barriers to implementing a suicide prevention protocol in primary care. The Consolidated Framework for Implementation Research (CFIR) (47–49) guided our exploration of the barriers, and facilitators’ participants faced implementing the suicide prevention protocol. The CFIR framework comprises five “determinant” domains that directly influence implementation outcomes, such as adoption, implementation, and sustainment of evidence-based interventions within the healthcare system (48). Phase 2 included qualitative matching of barriers to implementation strategies. We used the Expert Recommendations for Implementing Change (ERIC) CFIR tool (50, 51) to match the CFIR-identified barriers with practical implementation strategies. Phase 3 included quantitative Plan Do Study Act (PDSA) evaluation cycles (52) to test the acceptability and feasibility of relevant implementation strategies.

### 2.2 Setting

Our study was conducted with RAs and HCPs, respectively (clinical and non-clinical providers who are STOP implementers at the clinic), drawn from the INTREPID Lab (a team applying multidisciplinary approaches toward tobacco and vaping cessation, chronic disease prevention, and behavior change strategies) at CAMH and from 123 primary care clinics that operate the STOP program (43).

### 2.3 Participants and recruitment

#### 2.3.1 Eligibility

HCPs were eligible to participate if they worked in a team-based primary care clinic in Ontario (e.g., a family health team, community health center, or nurse practitioner led clinic), participating in the STOP program, and had experience with the suicide screening, assessments, and prevention protocols and willing to participate in all three phases of the study. STOP RAs were eligible if they had been trained on the suicide prevention protocol and willing to participate.

#### 2.3.2 Recruitment

To increase the likelihood of recruiting HCPs with previous exposure to the suicide prevention protocol, we utilized purposive sampling. This involved selecting individuals from clinics with a documented history of notably elevated suicidality prevalence. We identified clinics with significantly above-average prevalence by calculating a logit-transformed 95% confidence interval for the proportion within each clinic. Selection criteria included choosing clinics where the lower bound exceeded the overall STOP prevalence of 4.5%. Subsequently, CAMH research staff informed these clinics about the study via email, inviting them to participate. In addition, we advertised the study at the STOP program monthly meetings (approximately 75 attendees) and via an email to all STOP implementers (an estimated 1,200 individuals). HCPs interested in participating in the study, contacted CAMH research staff in order to enroll.

To recruit STOP RAs, we emailed the STOP manager, INTREPID Lab, with the project details and a request for RAs to contact the research coordinator if interested in participating in the study. RAs were asked to participate only in Phase 1 of the study, as they were not able to test Phase 3 implementation strategies.

The research coordinator conducted consent discussions via telephone or virtually through WebEx. In total, 11 HCPs who are STOP implementers from eight primary care clinics and 4 RAs from the INTREPID Lab who were trained in administering the suicide prevention protocol to patients enrolled in STOP consented to participate in the present study.

### 2.4 Procedures

Phase 1 elicited key barriers to the implementation of a suicide prevention protocol by conducting semi-structured interviews with HCPs and RAs via WebEx. Semi-structured interview guides were based on the CFIR domains (47). Specifically, we included questions regarding the characteristics of suicide screening, assessment protocol (e.g., evidence strength and quality, complexity), the outer setting (e.g., patient needs and resources), inner setting (e.g., compatibility of the protocol with existing programs, leadership engagement), the process used to implement the program (e.g., quality and extent of planning, engagement of key stakeholders), and characteristics of individuals involved (e.g., knowledge and attitudes; Supplementary material S1). The research coordinator (co-author AG), a PhD health researcher with advanced training and several years of experience in qualitative research methodology, conducted the interviews. Interviews ranging from 45 to 60 min in duration were audio recorded and transcribed verbatim. Identified barriers were mapped onto the CFIR constructs and domains. In addition, a brief sociodemographic survey was administered (e.g., gender, training, and role) through REDCap.

Phase 2 leveraged insights garnered from Phase 1 to design a menu of implementation strategies, with input from HCPs. The CFIR constructs and identified barriers were subsequently mapped to ERIC strategies (50, 51) that best addressed each CFIR barrier. Given that this tool usually suggests several implementation strategies for each barrier, a matrix was created, showing all the implementation strategies that can be used to address each barrier. Using the tool, we prioritized a list of ERIC implementation strategies for consideration. Data from these steps were used to create presentations to the research team for review and discussion. CFIR identified barriers, ERIC strategies were refined, and consensus discussions were held with HCPs, virtually using WebEx. HCPs were asked to rank and endorse the most relevant ERIC implementation strategies.

Phase 3 tested the endorsed implementation strategies, addressing key barriers. Employing the PDSA method, we asked HCPs to test the implementation strategies endorsed in Phase 2. The four prescribed steps of the PDSA guided the design and development (plan), the execution and the evaluation (Do), the analysis (Study), and reporting of the findings (Act) of these strategies (28, 31). For a given PDSA cycle, we assessed the feasibility and acceptability of the implementation strategy using the Acceptability of Intervention Measure (AIM) and the Feasibility Intervention Measure (FIM). The AIM (sample item, “The Evidence-Based Intervention [EBI] seems appealing to me”) and the FIM (sample item, “The EBI seems implementable”) are unidimensional measures comprising four items each with good structural validity, internal consistency reliability, and test–retest reliability. The outcome measures were scored on a five-point ordinal scale with answer choices ranging from “completely disagree” to “completely agree.” Outcome data were collected via a brief survey administered through REDCap. To maintain blindness, a separate database of these measures was generated without record ID or participant characteristics data. Development of each PDSA cycle took approximately 4 to 10 weeks.

### 2.5 Data analysis

Demographic data collected in Phase 1 were used to describe the sample and were presented in tabular format. Interviews, also conducted in Phase 1, were audio recorded and transcribed verbatim by a professional transcribing service. De-identified transcripts were analyzed using Rapid Qualitative Analysis (53), which involved deductive coding based on the CFIR domains and constructs. This rapid qualitative analysis approach is an applied method used to obtain actionable, targeted qualitative data on a shorter timeline than traditional qualitative methods (54). This data analysis approach was suitable as our interview protocols were highly structured, and data collection and analysis aimed to identify and broaden the understanding of key mechanisms, intervention elements, salient descriptors, facilitators, and barriers of a program to address time-sensitive research questions (55). Using questions from the CFIR-informed interview guides, we developed a structured template for summarizing verbatim transcripts that included identification of illustrative quotes. Summaries were subsequently consolidated into Excel matrices based on participant type to facilitate display of the data (56). These matrices were used to identify commonly identified categories of lessons learned about the barriers and facilitators of the implementation of a suicide prevention protocol in primary care (57). Subsequently, we used these categories of lessons learned to develop and refine a CFIR-based codebook displaying into which CFIR construct and domain the commonly identified lessons fell.

AIM and FIM items, collected in Phase 3, were summed up, with possible scores ranging from one to five for each scale, and higher scores indicate higher feasibility and acceptability, respectively. Descriptive statistics were computed using Microsoft Excel 2016.

### 2.6 Ethical considerations

The Research Ethics Board at the Center for Addiction and Mental Health approved the study (Protocol# 073/2022). Before conducting the interview, we explained the objectives of the study to participants and obtained written informed consent for their participation and audiotaping. The interviewer (AG) emphasized the optionality of taking part in the study. Furthermore, we informed participants of their right to withdraw from the study and stop the interview at any time. We did not compensate participants for their participation.

## 3 Results

Data collection took place between October 2022 and June 2023. We conducted 15 semi-structured interviews with 4 RAs and 11 HCPs. Participant characteristics are presented in Table 1. Transcripts from all 15 interviews served as the primary data source for Phase 1 of the study.

**Table 1:** Participant characteristics.

Participant/Site	INTREPID, CAMH	Primary care clinics (participating in STOP)
Interviewed (*n*)	4	11 (from 8 primary care clinics)
Organization type	STOP Program	Community Health Clinic, 9 (82%) Family Health Team, 1 (9%) Nurse Practitioner Led Clinic, 1 (9%)
Gender identity *n* (%)	Women, 4 (100%) Men, 0	Women, 8 (73%) Men, 3 (27%)
Job title *n* (%)	RAs, 4 (100%)	Nurse, 6 (55%) Nurse practitioner, 2 (18%) Outreach worker, 1 (9%) Therapist, 1 (9%) Health promoter, 1 (9%)
Practice location *n* (%)	Urban, 4 (100%)	Urban, 7 (64%) Rural, 3 (27%) Mixed urban rural, 1 (9%)
Training in suicide prevention protocol (hours) *n* (%)	4 to 10, 4 (100%)	More than 40, 6 (55%) 11 to 40, 3 (27%) Less than 10, 1 (9%) None, 1 (9%)

### 3.1 Phase 1: CFIR barriers

All participants identified the same key barriers and enablers leading to the amalgamation of findings from both groups into a single cohort for presentation and analysis. Below, we present the 10 key barriers and facilitators mentioned by the participants organized according to the CFIR domains.

#### 3.1.1 Intervention characteristics

##### 3.1.1.1 Design of the protocol and time required to implement in a busy practice setting

All participants (*n* = 15) thought that the protocol worked well with most patients, and it was well designed and clearly presented.

Yes, absolutely. I’m pretty comfortable with the way it’s designed in there. I do not find it too clunky. Yeah, there’s a good flow to it. IDI 09.

Some HCPs (*n* = 3) mentioned that it should be more culturally appropriate, and they adapted it to meet their patient population’s needs.

Well, it’s a good snapshot. It’s a quick, easy way to get a snapshot of somebody’s mental health…. And then newcomers… things that they do not sometimes understand what that is. Or even, you know, like mental health to them is not even… there’s no such thing. IDI 11.

#### 3.1.2 Inner setting

##### 3.1.2.1 Training and educational materials

All participants’ (*n* = 15) perception was that training in suicide prevention was a key enabler for implementation.

I feel somewhat confident that I can handle something that’s come up through the protocol questions that need attention. I’ve been doing this job for a while, about 10 years. I have background in addiction and mental health training.... I can gage on what level of… that person may need. IDI 08.

RAs mentioned that the training and access to procedural documents with specific instructions on administrating the protocol facilitated the use of the protocol.

And we did go through a training session, a couple of them actually, with one of the directors [name] who is a clinician. She’s a psychologist and a practitioner. And there was a fairly extensive SOP that we were able to read and reference back to that gave us pretty comprehensive instructions on how to conduct these PHQ-9 questions. IDI 03.

Two HCPs indicated that they received no formal training on administering the suicide prevention protocol, which impacted their level of confidence, potentially acting as a barrier to implementing a suicide prevention protocol in primary care.

I am at a loss to like… like I’m not really well trained in this, this is like beyond. …I’m also feeling like it’s all on me and sometimes I feel like that weight is on me and if something were to happen, I would feel responsible. Like I do not have the education for suicidal, you know, prevention and, yeah. Like I’ve taken courses to try to help but even though those courses are very I find surface level. IDI 07.

All HCPs mentioned that they felt strategies, such as training videos, webinars, lectures, and educational meetings, would facilitate implementation of a suicide prevention protocol.

I feel like having trainings around suicide it would be helpful… once a year or a couple times a year where people can just sign up and do a refresher… Even for me as an experienced practitioner, it’s always helpful to go to these things and remind myself to sort of think about these things. How we ask the questions. Are there new resources out there? Even simple training. Like it does not have to be a big thing but then sort of, you know, whether it be videos…IDI 06.

Having quick tools are always great. So if it’s like a flash card of … I do not know. You know, things that could be … Maybe even signage and posters that can be in our exam rooms. IDI 15.

##### 3.1.2.2 Available resources

Most HCPs (*n* = 9) described demands on their time as constraints to implementation and that as part of the smoking cessation program implementation required significant time and could be even lengthier if or when patients express suicide ideation.

They’re not quick conversations. Somebody just cannot kind of start producing their most fragile and difficult thoughts… and the provider’s saying, oh, I’ve got somebody else in 10 min later from now, hurry up with these, right? That’s a tough challenge. IDI 09.

Other factors that impede implementation are the lack of financial resources to pay for mental health services not covered the government run health plan (Ontario Health Insurance Plan (OHIP)) or healthcare insurance to enable patients’ access to mental health services.

Like that’s another barrier is that if psychotherapy was covered under OHIP that would be wonderful if, you know, psychologists could bill through that. Psychotherapists who are under the supervision of a psychologist, if they could bill through OHIP that would be wonderful, right? So yeah, a huge barrier to therapy is the cost. And usually insurance plans, like depending on who you are with – like if your employer has an insurance plan, like sometimes it’s like $500, which gives you two sessions, basically. If it’s $1,000, that’s good. That’s still is not a whole lot, so. IDI 014.

##### 3.1.2.3 Site characteristics

HCPs working in these team-based primary care settings mentioned that this model facilitated the implementation of the suicide prevention protocol.

So in a community health center setting, I think we do have the skills and the time to support folks around that. We have some access to counselors here if we need to connect folks. We have a case manager who does short-term stuff… can support folks.... people who are… more prone to feeling overwhelmed and potentially suicidal. I would imagine in some family health team setting it’d be very hard to do in a solo practice. But I think in some of the different models of care, I think primary care is probably a really good place to start, yes. IDI 05.

Some HCPs’ (*n* = 5) perception was that the capacity and readiness of their clinic to implement the suicide prevention protocol was low as 3 on a scale of 1 to 10. This was due, in part, to inadequately training providers to administer the protocol and provide appropriate care.

You know those types of screening questions… could definitely be integrated into any or most of our intake procedures. My impression is that at least most of the staff that aren’t comfortable working in the mental health field, which is probably at least 75% of the people at our clinic, struggle with these questions… is one area where we could do a lot of work, is build the capacity of staff to have conversations beyond these questions. I think there’s possibility for training and developing people. It’s [capacity to implement] very low in my mind. Like you want zero to 10? Like three. I’ll give you a three [readiness to implement]; same thing as the capacity. IDI 09.

##### 3.1.2.4 Leadership support

Most HCPs (*n* = 11) were not aware of any organizational goals and mandates to address suicide prevention, and their leadership was largely unfamiliar with the protocol as part of the STOP smoking cessation program. However, some providers mentioned that there were “in-house” mental health supports, and, as providers, they understood the importance of addressing suicide ideation/mental health as part of overall health even if not explicitly outlined in the organizational goals.

I think it’s probably less overtly named in the organizational goals of the strategic plan. Having said that, I think there is just an understanding that we work --you know, we have access to some mental health supports in-house and we all have a level of expertise with that and so it really works with our mandate to --you know, to see mental health as an important aspect of health overall. I think it’s just understood here you are, all you clinicians. But it’s not sort of embedded in anything that we do. IDI 15.

All participants (*n* = 15) indicated that leadership buy-in, support, and endorsement of explicit organizational goals are essential to promote suicide prevention in primary care.

It would involve some buy-in and as I said, I think there’s an awful lot of feeling that… now we have to add something else to what we are responsible for. I think it would probably have to come from within… and embedding it in some of the templates in the EMR. IDI 15.

#### 3.1.3 Outer setting

##### 3.1.3.1 Strong networks

All participants (*n* = 15) indicated that it was important to leverage networks for specialized patient care if needed. However, HCPs in rural settings noted that there were limited mental health resources. The lack of external networks for patient referral for further care is a barrier to the implementation. HCPs raised concerns about asking patients these questions if they were not going to connect patients to the care that they need.

At a provincial level, there needs to be a pathway where people can get connected. So the huge barrier is not enough services in, you know, primary care service, pre-hospital service, Ontario Health Insurance Plan (OHIP) covered mental health supporters. IDI 05.

##### 3.1.3.2 External policies and incentives

All participants (n = 15) thought that it would be appropriate to screen at-risk individuals and or populations for suicidal ideation in primary care settings but were not aware of any government-mandated guidelines, policies, or supports. HCPs noted that establishing government policies would require additional programs and financial incentives for providers.

I strongly encourage them to do more for programs and services, the health populations with depression. I know they have a program called [name]. It’s like online, they do that, it’s a great resource but it’s something that not everybody is willing to do unless they have a little bit of handholding to help them go through it. And the governments have also introduced certain things or they have these walk-in places now, you know, Canadian Mental Health walk-in counseling services where you just go and walk in. IDI 08.

That’s more of a touchy subject, I think, just because I know – I can only speak for us at the CHC model. There seems to always be demands but – this may sound ugly, but we are extremely unpaid compared to other people in the same professions… And most of us are here underpaid because we really believe in the model and we love what we do and we want to help the people, but I think there’s a lot of demands put on primary care... IDI 10.

#### 3.1.4 Characteristics of individuals

##### 3.1.4.1 Perceived value

All participants (*n* = 15) believed that asking questions about suicide ideation could save lives. These questions were important because it opened a door to having discussions about this critical issue, particularly in primary care settings, where they have an existing relationship and rapport with their providers. In addition, while provider needs can vary, there was consensus across groups that they appreciated having a suicide prevention protocol that would allow them to provide evidence-based care.

I think primary care’s the best place to start to address lots of things because there’s less stigma associated with going to your primary care provider and disclosing stuff. Hopefully, you know them so you feel more comfortable disclosing things. And then it [protocol] provides possibly for some folks a little bit of a launching pad to explore that further. IDI 05.

##### 3.1.4.2 Confidence to implement the protocol

Some participants (*n* = 6) expressed a lack of confidence because they were not clinicians and or believed that they had insufficient and or appropriate training leading to feelings of anxiety about asking patients about this sensitive issue. Moreover, some providers (*n* = 3) were not familiar with principles or rationale behind the inclusion of the protocol within a smoking cessation program. They mentioned that it would be beneficial to have training and a preamble that would provide a clear explanation of the relevance of screening for suicide ideation. In addition, they wanted more guidance for how to respond to positive screens and how to stratify risk and intervene appropriately.

So, you know, I know the PHQ is quite a standardized scale. I’ve never had training… I’ve just administered it. … So I feel like just maybe having more of a rationale or like a … Like a popup that says this is why we are asking this question. So that if a client gets defensive or starts wondering like why are you asking me all these questions, we are just better equipped to be like … To answer them in a way that might reassure them. And also reassure yourself, like – because of the purpose of asking these questions, even though my client just wants nicotine patches, we are asking these questions for these reasons. Disadvantage, off the bat, like I had mentioned, just that anxiety. Right? And again, might be related to lack of training, lack of context, anxiety about how the client will react about being asked these questions...IDI 12.

#### 3.1.5 Process

##### 3.1.5.1 Clinical champions

Clinical champions could facilitate the implementation of the suicide prevention protocol.

And especially with the pandemic, there’s been an increase in mental health issues, there’s been an increase in substance use issues. And I think it was highlighted by the providers…. And I pushed for it too. I had been asking for a while. I said, “Look, this is my speciality, this is what I like. And, you know, we have like a diabetes nurse educator. Why cannot we have a mental health nurse?” IDI 14.

I think it would probably have to come from within. I think it would be, you know, having a champion in the agency…IDI 15.

### 3.2 Phase 2: presented ERIC implementation strategies

CFIR-related barriers matched to CFIR domains and, subsequently, mapped to ERIC strategies were presented (Table 2) to HCPs. In consensus discussions, all HCPs endorsed three ERIC strategies as the most relevant for effective implementation of the suicide prevention protocol in primary care. The three ERIC strategies endorsed for testing in Phase 3 were: training (webinar), educational materials (infographic), and a preamble providing a rationale prior to asking the suicide screening questions.

**Table 2:** ERIC strategies to address implementation barriers.

CFIR domain and related barriers to implementation		ERIC strategies to address barriers	Specific action(s)
Domain	Related barriers		
Intervention characteristics	Stakeholders believe that the innovation is complex based on their perception of duration, scope, and number of steps needed to implement.	Conduct ongoing training	Plan for and conduct training in the innovation in an ongoing way for all individuals involved with implementation and users of the innovation, e.g., clinicians, implementation staff, practice facilitators.
Outer setting	The organization is not well networked with external organizations.	Build a coalition	Recruit and cultivate relationships with partners in the implementation effort.
	External policies, regulations (governmental or other central entity), mandates, recommendations or guidelines do not exist.	Involve executive boards Build coalition Alter incentive/allowance structures.	Involve existing governing structures (e.g., boards of directors, medical staff boards of governance) in the implementation effort, including the review of data on implementation process. Recruit and cultivate relationships with partners in the implementation effort. Work to incentivize the adoption and implementation of the innovation.
Inner setting	The social architecture, age, maturity, and size of an organization hinders implementation.	Assess for readiness and identify barriers and facilitators Change physical structure and equipment.	Assess various aspects of an organization to determine its degree of readiness to implement, barriers that may impede implementation and strengths that can be used in the implementation effort. Evaluate current configurations and adapt, as needed, the physical structure and/or equipment to best accommodate the targeted innovation.
	There is little capacity for change, low receptivity, and no expectation that use of the innovation will be rewarded, supported, or expected.	Assess for readiness and identify barriers and facilitators	Assess various aspects of an organization to determine its degree of readiness to implement, barriers that may impede implementation, and strengths that can be used in the implementation effort.
	Stakeholders do not see the current situation as intolerable or do not believe they need to implement the innovation.	Identify and prepare champions.	Identify and prepare individuals who dedicate themselves to supporting, marketing, and driving through an implementation, overcoming indifference or resistance that the intervention may provoke in an organization.
	There are few tangible and immediate indicators of organizational readiness and commitment to implement the innovation.	Assess for readiness and identify barriers and facilitators	Assess various aspects of an organization to determine its degree of readiness to implement, barriers that may impede implementation, and strengths that can be used in the implementation effort.
	Key organizational leaders or managers do not exhibit commitment and are not involved, nor are they held accountable for implementation of the innovation.	Involve executive boards Identify and prepare champions.	Involve existing governing structures (e.g., boards of directors, medical staff boards of governance) in the implementation effort, including the review of data on implementation process. Identify and prepare individuals who dedicate themselves to supporting, marketing, and driving through an implementation, overcoming indifference or resistance that the intervention may provoke in an organization.
	Resources (e.g., money, physical space, dedicated time) are insufficient to support implementation of the innovation.	Fund and contract for the innovation.	Governments and other payers of services issue requests for proposals to deliver the innovation, use contracting processes to motivate providers to deliver the innovation, and develop new funding formulas that make it more likely that providers will deliver the innovation.
Characteristics of individuals	Stakeholders are not familiar with facts, truths, and principles about the innovation.	Conduct educational meetings Develop educational materials Identify and prepare champions	Hold meetings targeted toward educating multiple stakeholder groups (i.e., providers, administrators) about the innovation and/or its implementation. Develop and format manuals, toolkits, and other supporting materials to make it easier for stakeholders to learn about the innovation and for clinicians to learn how to deliver the innovation. Identify and prepare individuals who dedicate themselves to supporting, marketing, and driving through an implementation, overcoming indifference or resistance that the intervention may provoke in an organization.
	Stakeholders do not have confidence in their capabilities to execute courses of action to achieve implementation goals.	Conduct ongoing training Make training dynamic	Plan for and conduct training in the innovation in an ongoing way for all individuals involved with implementation and users of the innovation, e.g., clinicians, implementation staff, practice facilitators. Vary the information delivery methods to cater to different learning styles and work contexts, and shape the training in the innovation to be interactive.
Process	Individuals acting as champions who support, market, or ‘drive through’ implementation.	Identify and prepare champions.	Identify and prepare individuals who dedicate themselves to supporting, marketing, and driving through an implementation, overcoming indifference or resistance that the intervention may provoke in an organization.
	Implementation activities are not being done according to plan.	Conduct ongoing training	Plan for and conduct training in the innovation in an ongoing way for all individuals involved with implementation and users of the innovation, e.g., clinicians, implementation staff, practice facilitators.

### 3.3 Phase 3: acceptability and feasibility of endorsed implementation strategies

HCPs endorsed the following top three ranked ERIC implementation strategies matched to the CFIR barriers: a webinar, adding a preamble to the STOP program portal prior to the PHQ-9 suicide prevention protocol questions, and an infographic. These strategies address the CFIR barriers related to stakeholders’ familiarity with the principles of the protocol and their confidence in their capabilities to achieve implementation goals. These barriers matched to the domain “characteristics of individuals.”

In PDSA cycles, HCPs reviewed and tested the selected implementation strategies. HCPs attended and reviewed the training webinar titled “When contact save lives: Strategies for suicide prevention in primary care,” the content of which was prepared by co-investigators RD and AC and delivered by co-investigators PS and AC on 15 May 2023. This 1-h interactive training session was included in the educational rounds monthly webinar series for healthcare providers across all disciplines, to enhance knowledge and skills organized by the INTREPID Lab TEACH team. The webinar was widely advertised by email and attended by 99 healthcare providers, including the study participants. The webinar was accredited, and participants were able to receive a credit for their attendance. The recorded webinar can be found on YouTube: https://youtu.be/h40_GbHaQRE. HCPs also reviewed the preamble or rationale “The following questions will ask about the patient’s mood. Identifying individuals with low mood and providing support and resources can help address challenges and increase the chances of a successful quit attempt,” which was prepared by the co-investigators and the STOP team and added to STOP portal on 16 April 2023. In addition, HCPs reviewed the infographic which was created, adapting content from mental health/suicide prevention sources (58–66), encapsulating the webinar content, and incorporating input from the expertise of the research team in mental health. The infographic (Supplementary material S2) was shared with the HCP study participants on 29 May 2023.

In the PDSA cycle, HCPs subsequently evaluated the strategies using the Acceptability of Intervention Measure (AIM) and the Feasibility of Intervention Measure (FIM), to ascertain perceptions of these leading indicators of the most relevant implementation strategies. Eleven HCPs (100%) completed the webinar, preamble, and infographic surveys. Participants endorsed high acceptability and high feasibility of the implementation strategies. Table 3 presents the descriptive statistics on AIM and FIM scores.

**Table 3:** Summary of outcome data.

	Webinar (*n* = 11)			Preamble (*n* = 11)			Infographic (*n* = 9)		
	Mean (SD)	L95%	U95%	Mean (SD)	L95%	U95%	Mean (SD)	L95%	U95%
Acceptability of Intervention Measure (AIM)	4.55 (0.55)	4.22	4.87	4.34 (0.55)	4.02	4.67	4.55 (0.49)	4.26	4.83
Feasibility of Intervention Measure (FIM)	4.64 (0.67)	4.24	5.03	4.45 (0.62)	4.09	4.82	4.39 (0.63)	4.02	4.76

## 4 Discussion

Our findings demonstrate shared factors that influence the implementation of a suicide prevention protocol within an established smoking cessation program in primary care in Ontario, Canada, including training, time constraints, leadership engagement (inner setting), collaborative networks, policies, and financial incentives (outer setting). Most HCPs believed that training and educational materials (webinar, preamble, and infographic) could help address barriers to implementation providers experienced in their clinics (inner setting).

In common with other research studies, we found that the lack of appropriate training, knowledge, and the confidence to assess suicidal thoughts were significant challenges to implementing effective suicide prevention in primary care settings (67–69). Webinar training and educational materials were endorsed and evaluated by study HCPs as highly acceptable and feasible implementation strategies to influence effective implementation of suicide prevention protocol. Studies confirm that training can change provider attitudes, improve knowledge, and increase confidence in diagnosing and treating their mental health patients (70, 71). However, one study suggested that educating primary care providers is beneficial but on its own it is insufficient. Such improved liaison between mental health services and primary care is needed for effective suicide prevention (31).

The study findings highlighted the need for stronger mental health networks or coalitions, funding resources, supportive policies, and insurance coverage. Our findings are consistent with other studies that show mental health networks poorly integrated with primary care, lacking standardized specific approaches to mitigating suicide risk in their patients often overlook at-risk individuals (4, 31, 67, 72). In the absence of sufficient evidence for universal assessment of suicide prevention, Canada has developed public health policy that prioritizes suicide prevention among high-risk groups in primary care (73–75). Moreover, the Public Health Agency of Canada, recognizing that suicide remains a leading cause of death and that the rate of suicide ideation has been increasing, has committed to funding suicide prevention toward creating a national network of crisis services (CSC) (4, 8, 76, 77).

Participants were aware that there are no explicit government mandates about suicide prevention, but they all agreed in the value of having a suicide prevention protocol and the importance of screening high-risk patients for suicide ideation in primary care. Studies confirm that primary care is crucial for suicide prevention because the trust and rapport providers develop with patients over time in this setting were key to addressing this sensitive and critical public health issue (28–31, 67). Furthermore, it is recommended that primary care clinicians consider screening for suicidal behavior among smokers because the complex interplay between smoking and suicidal behavior is important (78). A meta-analysis confirms that because of the prospective relationship between smoking and increased risk of suicidal thoughts and behaviors (death by suicide, ideation, or attempts), smoking should be included in risk scales as a useful and easy item to evaluate suicide risk (79).

Important challenges faced by study participants included limited time to assess suicidal thoughts. Completing suicide ideation assessments within the STOP smoking cessation program was especially concerning as it requires significant amount of time for both providers and patients. This is consistent with other studies that describe time constraints as barriers to the implementation of suicide prevention practices (31, 67, 68). Participants mentioned that there were no explicit clinic goals for suicide prevention, and that greater leadership engagement and support and the presence of a champion would improve implementation of the suicide prevention protocol. Evidence suggest that leadership has a central role to reduce suicide for people under its care by emphasizing suicide prevention as a critical patient safety issue and supporting staff members providing care for individuals who express suicidal behaviors (69).

There are many challenges to the effective implementation of suicide prevention in primary care where most patients who subsequently die by suicide are observed (2, 69). A well-structured, evidence-based suicide prevention protocol (intervention characteristics) along with appropriate training and educational materials (inner setting) that improve provider knowledge and confidence (characteristics of individuals) has the potential of improving the implementation of suicide prevention in primary care. However, leadership of a healthcare organization (inner setting) and stronger mental health networks, policies, and incentives (outer setting) are also required to address this complex and sensitive public health issue.

### 4.1 Strengths and limitations

The use of the CFIR framework and ERIC compilation were key strengths of this study, given the flexibility and adaptability of the framework identifying key influences on implementation from stakeholders’ perspectives (53) and the benefits of the compilation in prospectively supporting consideration of a broad array of strategies (80). Another important strength of this project was the three-phase mixed-method sequential exploratory design. This design allowed us to identify barriers to effective implementation of an intervention and use the barriers to select implementation strategies ranked by relevance in consensus discussions, as well as to test the main implementation strategies and evaluate their feasibility and acceptability in Plan Do Study Act (PDSA) cycles (80, 81).

Geographical diversity in the study sample was another strength. Participant data from rural, urban, or mixed rural–urban settings indicated that the suicide prevention protocol was well designed and appropriate for patients from diverse geographic locations, regardless of age, gender, and economic status. However, it needed to be adapted based on patients’ language and literacy levels.

Limitations include the fact that qualitative data cannot determine the effectiveness of the intervention on measurable outcomes such as provider confidence. While the targeted participants (STOP implementers) were arguably, the most appropriate individuals within the clinics to respond to the types of questions asked the purposive sample might not have been a representative sample possibly, because those who participated were champions of implementing suicide prevention in primary care and more likely to participate in the surveys and interviews. Although the small sample size (11 HCPs out of 1,200 HCPs from primary care clinics) may not be a representative sample, qualitative research does not rely, and quantitative evaluation rarely does, on large and statistically representative samples for its credibility. However, site selection and how participants were identified and recruited to participate are essential to contextualize the study findings (82). Purposive sampling was used to recruit participants who had knowledge and experience with the suicide prevention protocol, allowing for information-rich cases for an in-depth study (83). In addition, the small sample of people interviewed may have hindered achieving data saturation and possibly the identification of other factors that influenced implementation. The small sample also limited the ability to stratify the findings by demographics or other variables of interest. Moreover, on a conceptual level, while CFIR barriers, at the system and structural levels, were identified by HCPs, the strategies they endorsed were educational and or informational. This may be a product of who the interviewees were, and future directions may consider the perspectives of other stakeholders. Finally, there can be ethical, technical, and social challenges conducting qualitative research in a virtual environment (84). However, participants had access to a computer with good quality internet connection and a quiet and private space for the interview, and they were comfortable sharing their experiences which contributed to the richness of the data generated.

## 5 Conclusion

The study identified barriers related to primary care inner settings and provider characteristics using CFIR domains and ERIC implementation strategies to address these barriers. PDSA cycles confirmed the acceptability and feasibility of selected implementation strategies. These results offer evidence-based solution to increase the use of a suicide prevention protocol in smoking cessation programs delivered in primary care settings. Future efforts should track implementation of these strategies, measure outcomes, including provider confidence, self-efficacy, and knowledge, and patient outcomes.

## Data availability statement

The raw data supporting the conclusions of this article will be made available by the authors, without undue reservation.

## Ethics statement

The studies involving humans were approved by the Research Ethics Board at the Center for Addiction and Mental Health (Protocol# 073/2022). The studies were conducted in accordance with the local legislation and institutional requirements. The participants provided their written informed consent to participate in this study.

## Author contributions

NM: Conceptualization, Funding acquisition, Supervision, Writing – original draft. AG: Formal analysis, Investigation, Project administration, Writing – original draft. AdC: Conceptualization, Funding acquisition, Writing – review & editing. RD: Conceptualization, Funding acquisition, Writing – review & editing. LZ: Conceptualization, Funding acquisition, Writing – review & editing. JZ: Conceptualization, Funding acquisition, Writing – review & editing. BO’N: Conceptualization, Funding acquisition, Writing – review & editing. SL: Conceptualization, Funding acquisition, Writing – review & editing. NT: Conceptualization, Funding acquisition, Writing – review & editing. AlC: Conceptualization, Funding acquisition, Writing – review & editing. SK: Conceptualization, Funding acquisition, Writing – review & editing. PS: Conceptualization, Funding acquisition, Writing – review & editing.

## Footnotes

^1^The suicide-related thoughts and behavior terms used in this study are defined as follows: Suicide is an act resulting in death which is initiated and carried out by an individual to the end of the action, with the knowledge of a potentially fatal result, and in which intent may be ambiguous or unclear, may involve the risk of dying, or may not involve explicit intent to die. Suicide ideation is to think of suicide with or without suicidal intent, or hope for death by killing oneself, or state suicidal intention without engaging in behavior. Suicide-related behaviors are defined as a self-inflicted, potentially injurious behavior which can result in no injuries, injuries, or death. Suicide-related behaviors comprise self-harm, suicide attempts, and suicide. Self-harm is a non-fatal act in which a person harms himself or herself intentionally, with varying motives including the wish to die. Suicide attempt is an act, in which a person harms himself or herself, with the intention to die and survive.
