# Supplementary material for: Prototyping the implementation of a suicide prevention protocol in primary care settings using PDSA cycles: a mixed method study
Source: Front Psychiatry. 2024 Jan 25;15:1286078. doi: 10.3389/fpsyt.2024.1286078 (PMC10850298; doi:10.3389/fpsyt.2024.1286078)
Supplement: Supplementary file 1 [file Data_Sheet_1.PDF]

Supplementary Material

Prototyping the implementation of a suicide prevention protocol in primary care settings using PDSA cycles: A mixed method study

Nadia Minian, Allison Gayapersad, Adina Coroiu, Rosa Dragonetti, Laurie Zawertailo, Juveria Zaheer, Braden O'Neill, Shannon Lange, Nicole Thomson, Allison Crawford, Sidney Kennedy, Peter Selby

*** Correspondence:** Nadia.Minian2@camh.ca

1 Supplementary File 1

Supplementary file 1 - CFIR interview questions - Health Care Providers

I INTERVENTION CHARACTERISTICS

1) What do you know about the suicide prevention protocol?

- What do you like about it? What do you dislike?
- Has your clinic adapted the protocol for use in your clinic? If so, could you describe some of the adaptations that were made? If not, are there any adaptions that you would like to see?
- Are people in your clinic using the protocol?
- What are the advantages and disadvantages of this protocol?
- Do you think this protocol is useful for all patients with suicide ideation? (Probe for diversity: age, ethnicity, SES)
- What changes would you suggest we make to the protocol?
- Do you feel that the protocol is well designed and packaged, including how it is assembled, bundled, and presented?
- If you have not seen the suicide prevention protocol, what do you think would be important/useful for a suicide prevention protocol to include?

II OUTER SETTINGS

2) What can you tell us about the need to implement the suicide prevention protocol now/at the current time in your clinic?

- What do you think about addressing suicide prevention in primary care sites in Ontario?
- How well do you think the protocol will meet the needs of patients served by your organization? (Probe for diversity: age, ethnicity, SES)
- How do you think the individuals served by your organization will respond to being asked about suicide ideation/suicidal thoughts? (Probe for diversity: age, ethnicity, SES)
- Are there other organizations that currently address suicide prevention? Is there a collaboration (or a shared vision) to address suicide prevention more widely across the province?
- What is the provincial and/or federal mandates (or a push) for primary care to address suicide?

III INNER SETTINGS

3) What is the level of support to implement the suicide prevention protocol within your organization?

- How does suicide prevention fit with the goals/mandates of your organization?
- According to you, what are the impressions of the protocol from management? Service providers?
- How can the protocol be integrated with existing protocols/frameworks of care delivery?
- What is the overall capacity to support the protocol? (rate on a scale from 0-10)

- How can we encourage providers to share concerns, successes, and failures with using the protocol with peers and stakeholders?
- How would you rate organizational readiness for implementing the protocol (rate on a scale from 0-10)
- What type of resources might be necessary to successfully implement the protocol in your clinic?
- Has there been any recent practice guidelines implemented in your clinic?
  - What are the lessons learned from prior successes & failures?

IV CHARACTERISTICS OF INDIVIDUALS

4) -What are the key provider/implementer characteristics that would contribute to the successful implementation of this suicide prevention protocol?

- What are your thoughts about the suicide prevention protocol being delivered in your setting?
- How confident are you that you will be able to successfully implement the suicide prevention protocol?
- How confident do you think your colleagues feel about implementing the suicide prevention protocol?
- How knowledgeable are the providers that work in your clinic relative to suicide prevention interventions?
- Do you think following the suicide prevention protocol could save lives? (Probe for diversity)
- How can we facilitate provider confidence about using the suicide prevention protocol?
- How can we motivate providers to use/adhere to the suicide prevention protocol (e.g., systematic screening, etc)?
- How can we track/record provider needs when implementing the protocol?
  - What kind of support should be available to providers?

V PROCESS

5) What is the actual process that needs to be set in place for the organization to adopt the suicide prevention protocol?

- What kind of strategies can we use to engage organizations and providers to adopt the protocol?
- Would a webinar about the benefits of implementing the protocol help?
  - If so, should it be targeted to leadership or to HCPs?
  - How about forming a working group within your organization?
- Are there influencers/stakeholders who can leverage their status to promote the adoption of the protocol?
- Who are the official/formal implementers in the organization?
- Who will/could champion the protocol within the organization?

Supplementary file 1 - CFIR interview questions – Research Assistants

I INTERVENTION CHARACTERISTICS

1) What do you know about the suicide prevention protocol?

Probes:

- What do you like about it? What do you dislike?
- What are the advantages and disadvantages of this protocol?
- Would you suggest making any changes to the protocol?

II OUTER SETTINGS

2) What can you tell us about the need to implement the suicide prevention protocol?

Probes:

- What do you think about addressing suicide prevention in primary care sites in Ontario?
- How well do you think the protocol will meet the needs of patients seen in primary care?
- How do you think the patients seen in primary care will respond to being asked about suicide ideation?

III INNER SETTINGS

3) What is the level of organizational support to implement the suicide prevention protocol in primary care?

Probes:

- How can we encourage providers to share concerns, successes, and failures with using the protocol with peers and stakeholders?
- How would you evaluate organizational readiness for implementing a suicide prevention?
- What type of resources might be necessary to successfully implement the protocol in primary care?

IV CHARACTERISTICS OF INDIVIDUALS

4) What are the key provider/implementer characteristics that would contribute to the successful implementation of the suicide prevention protocol in primary care settings?

Probes:

- How do you feel about the suicide prevention protocol being used in primary care settings?
- How confident are you that you will be able to successfully use the protocol in primary care?
- How confident do you think your colleagues are about implementing the protocol in primary care?
- How can we facilitate provider confidence about using the protocol?
- How can we motivate providers to adhere to the protocol (e.g., systematic screening, etc)?
- How can we track/record provider needs when implementing the protocol?
    - What kind of support should be available to providers?

V PROCESS

5) What is the actual process that needs to be set in place for the organization to adopt the suicide prevention protocol?

Probes:

- What kind of strategies can we use to engage organizations and/or providers to adopt the protocol? –
    - What would help to adopt it?
    - What supports do you think you would benefit from?
    - Would a webinar about the benefits of implementing the protocol help? If so, should it be targeted to leadership or to HCPs?
    - How about forming a working group within your organization?
- Are there influencers/stakeholders who can leverage their status to promote the adoption of the protocol?

2 Supplementary File 2

Supplementary Figure 1. Infographic: Prevention Suicide in Primary Care – Risk Assessment and Possible Interventions.

camh
Centre for Addiction and Mental Health

Preventing Suicide in Primary Care

Risk Assessment and Possible Interventions

General information only: Please consider the needs of diverse peoples, including First Nations, Inuit, Métis, people who identify as 2SLGBTQ+, and immigrant, refugee, ethnocultural and racialized groups[1].

Patients who express thoughts of suicide or self-harm:

Conduct risk assessment with patients to evaluate if "at risk for suicide".

Assess Risk Factors

Sociodemographic
People who die by suicide in Canada are more likely to be[2-3]:

- Male (Age 35 - 64)
- Single, live alone
- Unemployed
- Alcohol and substance misuse

Psychological
Treating mental illness can reduce risk of self-harm and suicide[2-5].

- Depression
- Anxiety

Psychosocial
Addressing psychosocial problems can contribute to the building of resilience in the face of challenging life circumstances[2-5].

- Recent loss
- Lack of support
- Isolation

History
Finding out about a patient's past can reveal further risk factors, such as[2-5]:

- History of mental illness
- Survivors of suicide loss & suicide attempt
- History of abuse or trauma
- History of Chronic disease

Self-Harm Plans
Asking about suicide plans can be helpful. Consider introducing the subject gently, starting with questions like[5-9]:

- How are you doing?
- I am concerned about you! Are you thinking about suicide?

* Ask about suicide plans if deemed "at risk". Be direct, assertive, supportive!

1. Have you made a plan about how you might end your life?
2. Have you taken steps to carry out your plan to end your life?
3. Is there a date or time you intend to carry out your plan?

The 3 questions above will help you determine if the patient is at imminent risk of suicide.

Assess Protective Factors

Several protective factors have been identified, that can mitigate risks, if present[2-5]:

- Positive social support
- Strong religious faith
- Children at home
- Sense of responsibility
- Problem solving skills

Loss of protective factors can increase risk for the patient, for example if a partner and children leave the patient.

Estimate Risk Level[5-7]

Assess level of risk based on suicide plan, taking into account risk and protective factors.

Note: Difficult to reliably predict risk! Need to consider changes from patient's baseline.

Low risk ⟷ **High risk**

Low risk	Moderate risk	High risk
No plan and no serious risk.<br><br>Engages with treatment.	Low intent but has serious risk factors.<br><br>Ambivalent, confused, curious about suicide. May have suicide plan but have protective factors.	Has a well thought out plan or recent suicidal behaviours. Other risk factors (e.g. impulsivity, substance use, loss of rational thinking).<br><br>Hopeless, helpless, resigned. No engagement with treatment.

Possible Intervention[5-7]

Validate patient's feelings. Express concern, caring and show support.

Acknowledge that thoughts of suicide are common/frequent.

Consider whether the patient is willing and able to engage with a management plan.

Collaboratively address risk factors that can be modified.

Low risk:

Manage in primary care: Educate patient on depression. Start treatment.

Encourage patient to reduce/eliminate alcohol/substance use. Ask patient to remove, lock up, or have someone hold, any lethal weapons or dangerous amounts of medication.

Provide/refer for counselling. Arrange weekly appointments.

Encourage the patient that if anything changes, to contact the distress line (**Talk suicide 1.833.456.4566 or text 45645**), or **call 9-1-1** or report to the nearest Emergency Department, and involve a supportive person/individual.

Moderate risk:

Request an urgent psychiatric consultation (within 24 to 48 hours).

Ask patient to remove, lock up, or have someone hold, lethal weapons and medications. Ask about friends or family who could help.

See patient the next day to reassess, and then see patient frequently (based on level of suicidal intent) until psychiatric consultation takes place or their suicide risk returns to baseline.

Encourage the patient that if anything changes to contact the distress line: (**Talk suicide 1.833.456.4566 or text 45645**), **call 9-1-1** or report to the nearest Emergency Department.

High risk:

Have the patient transferred immediately to the nearest emergency room.

Consider the need for certification under the mental health act (consider severity; patient's insight; risk of elopement; level of cooperation).

Adapted from: BMJ 2017; 356 doi: https://doi.org/10.1136/bmj.j1128 (Published 30 March 2017) Cite this as: BMJ 2017;356:j1128

camh
Centre for Addiction and Mental Health

Help is available

- **Call 9-1-1**
- Canada Suicide Prevention Service - **Talk suicide: 1-833-456-4566 [Available: 24/7/365] or Text 45645 (4:00pm - Midnight ET)**
- Kids help phone: Call 1-800-668-6868 (toll-free) or text CONNECT to 686868. (Available: 24/7)
- Visit the Kids Help Phone website for online chat support or to access online resources for children and youth: https://kidshelpphone.ca/
- Distress and crisis Ontario member locations and contact information near you: https://www.dcontario.org/services/ and other resources: https://www.dcontario.org/resources/usefulresources/
- Trans Lifeline: 1-877-330-6366
- Hope for Wellness Help Line: 1-855-242-3310. Online chat: www.hopeforwellness.ca
- Indian Residential Schools Crisis Line: 1-866-925-4419
- CONTACT A CRISIS CENTRE NEAR YOU: Canadian Association for Suicide Prevention: www.suicideprevention.ca/need-help
- ADDITIONAL RESOURCES: www.canada.ca/en/public-health/services/suicide-prevention.html

1. Ontario Hospital Association and Canadian Patient Safety Institute. (2021). Suicide Risk Assessment Toolkit: A Resource for Healthcare Workers and Organizations. https://www.healthcareexcellence.ca/media/cidj0qr0/mhcc-cpsi-suicide-risk-assessment-toolkit-en-final-ua.pdf
2. Suicide: risks and prevention: https://www.canada.ca/en/public-health/services/suicide-prevention/suicide-risks-prevention.html
3. Suicide in Canada: Key statistics (infographic): https://www.canada.ca/en/public-health/services/publications/healthy-living/suicide-canada-key-statistics-infographic.html
4. Centre for Addiction and Mental Health - Suicide: https://www.camh.ca/en/health-info/mental-illness-and-addiction-index/suicide
5. Exploring thoughts of suicide. BMJ 2017; 356 doi: https://doi.org/10.1136/bmj.j1128 (Published 30 March 2017)
6. Nicotine Dependence Clinic. Resources for Providers. Working with patients presenting suicide ideation protocol: https://www.nicotinedependenceclinic.com/en/teach/Documents/Working%20with%20Patients%20Presenting%20Suicidal%20Ideation.pdf
7. Centre for Addiction and Mental Health. Suicide Risk: Managing Suicidality: https://www.camh.ca/en/professionals/treating-conditions-and-disorders/suicide-risk/suicide---managing-suicidality
8. Buddy Up ‘ “How are you REALLY doing?” : www.buddyup.ca
9. Just ask: https://www.cpslmind.org.uk/stop-suicide-just-ask-campaign-launches-for-world-suicide-prevention-day/
